# Evaluating medical education in Brazil: analysis of the National Student Performance Exam 2023

**DOI:** 10.3389/fmed.2025.1679924

**Published:** 2025-10-23

**Authors:** Rogerio Rufino, Mario Fritsch Toros Neves, Adauto Dutra Moraes Barbosa, Andrea Povedano, Alberto Schanaider

**Affiliations:** ^1^Faculty of Medical Sciences, Universidade do Estado do Rio de Janeiro (UERJ), Rio de Janeiro, Brazil; ^2^Biomedical Center, Universidade do Estado do Rio de Janeiro (UERJ), Rio de Janeiro, Brazil; ^3^Faculty of Medicine, Universidade Federal Fluminense (UFF), Niterói, Brazil; ^4^Faculty of Medicine, Universidade Federal do Estado do Rio de Janeiro (UNIRIO), Rio de Janeiro, Brazil; ^5^Faculty of Medicine, Universidade Federal do Rio de Janeiro (UFRJ), Rio de Janeiro, Brazil

**Keywords:** educational assessment, medical education, public policy, regional inequalities, ENADE

## Abstract

**Background:**

The evaluation of medical education in Brazil relies on instruments such as the National Student Performance Exam (ENADE) and the Preliminary Course Concept (PCC), which guide regulation and funding.

**Objectives:**

To analyze national data from the 2023 ENADE for the medical program, describing variations by administrative category and region, and to discuss implications for building a fairer assessment model aligned with the principles of the Brazilian Unified Health System (SUS).

**Methods:**

A descriptive study using consolidated data from 309 Medical Programs participating in ENADE 2023. Mean scores and standard deviations for the continuous PCC (scale 0–5) were calculated by institutional category (federal, state, municipal public institutions, and private with or without profit) and geographic region.

**Results:**

Federal public institutions showed a mean PCC of 2.82 ± 0.38; state, 2.74 ± 0.42; and municipal, 2.65 ± 0.45. Private for-profit institutions had 2.72 ± 0.48, and non-profit institutions 2.85 ± 0.42. The Indicator of Difference Between Observed and Expected Performance (IDOEP) component, which accounts for 35% of the PCC, was higher in for-profit private institutions (3.70 ± 0.68) compared to public ones (2.65 ± 0.50), reflecting limitations in adjusting for socioeconomic intake profiles. Student perception scores were also higher in private institutions (3.85 ± 0.60) than in public ones (3.00 ± 0.55). Regionally, PCC means were higher in the South (3.45 ± 0.40) and Southeast (3.35 ± 0.45) than in the Northeast (2.85 ± 0.55) and North (2.70 ± 0.60).

**Conclusion:**

Results suggest that the current ENADE/PCC model may mask structural and regional inequalities, favoring institutional strategies focused on large-scale enrollment with lower admission requirements.

## Introduction

### History of medical education in Brazil and the development of National Curriculum Guidelines

Medical education in Brazil has colonial roots, with the first medical schools created in Salvador and Rio de Janeiro in 1808, tied to the arrival of the Portuguese royal court (1). For over a century, training followed a biomedical, hospital-centered model based on isolated disciplines, with limited integration between theory and practice or alignment with population health needs. While several reforms were proposed throughout the 20th century, only in the late 1990s did a consistent national movement for broader, more systematic change emerge. The National Education Guidelines and Framework Law (LDB No. 9.394/96) (2) laid the foundation for reforming curricula across higher education in Brazil, enabling the development of National Curriculum Guidelines (DCNs) for undergraduate Medical Programs (3).

The first Medical DCNs, approved in 2001 (3), defined the graduate profile as a generalist, critical, reflective, and humanistic professional, capable of working according to the principles of the Brazilian Unified Health System (SUS) (4). These guidelines sought to overcome the traditional Flexnerian model, promoting active teaching methodologies, greater integration between teaching and service, and a focus on population health needs (3, 5).

In 2014, updated Medical DCNs were approved (CNE/CES Resolution No. 3/2014) (6), incorporating critiques and lessons accumulated over more than a decade. These guidelines reaffirmed the commitment to competency-based education, lifelong learning, and the social responsibility of training institutions (6).

Despite this progress, implementation remains uneven. Many schools face challenges related to rigid curricula, insufficient funding, inequality among institutions, and lack of qualified practice settings (7, 8). Public initiatives like the More Doctors Program (PMM) (9) aimed to strengthen medical education by expanding undergraduate and residency training and improving regional distribution of professionals.

In this context, external quality assessment of Medical Programs plays a central role in regulation and accountability. Since 2004, the National Student Performance Exam (ENADE) (10) has been the primary instrument for evaluating student performance in line with the DCNs. ENADE results are used to calculate the Preliminary Course Concept (PCC), which informs course recognition and renewal decisions by the Ministry of Education (INEP) (10). However, little is known about whether national large-scale evaluations such as ENADE and PCC adequately reflect these structural inequalities or support curricular improvement aligned with the DCNs.

In 2025, the National Medical Students Assessment Exam (ENAMED) (11) will replace National Student Performance Exam (ENADE), aiming to implement an annual evaluation of medical programs. However, it remains uncertain whether ENAMED will address the current misalignment between assessments and the 2014 National Curriculum Guidelines (DCNs), which emphasize competencies for work within the Unified Health System (SUS). Although ENAMED may allow new graduates to use scores to apply for medical residency, the continuity of ENADE’s evaluation criteria has not been clarified.

The 2014 National Curriculum Guidelines (CNE/CES Resolution No. 3/2014) (12) marked a significant advancement by specifying the competencies expected of medical graduates and promoting integration between teaching, health services, and communities. Yet, their broad language allows varied institutional interpretations, complicating standardization. Implementation has also been hindered by unequal institutional capacity, insufficient practice settings, and limited faculty training in active methodologies. Challenges persist in integrating education with the SUS due to administrative barriers and fragile partnerships, such as the Organizational Contract for Public Teaching-Health Action (COAPES) (13), which often lack safeguards for pedagogical quality in hiring clinical preceptors (14). Despite promoting formative, competency-based evaluation, summative exams remain dominant, and limited investment in faculty development contributes to the persistence of traditional, lecture-based approaches.

## Objectives

This study aims to analyze how medical schools in Brazil are evaluated through the 2023 edition of the National Student Performance Exam (ENADE) (10) and the associated Preliminary Course Concept (PCC). It investigates the structure and weighting of key indicators, particularly the ENADE score, the Indicator of Difference Between Observed and Expected Performance (IDOEP) (10), and student questionnaire components. The analysis also examines disparities between public and private institutions regarding infrastructure, faculty qualifications, employment models, and student perceptions. Additionally, the study highlights methodological limitations of the current evaluation model, including the absence of on-site visits, statistical modeling assumptions, and reliance on subjective self-reported instruments.

## Methods

### Study design

This study is a documentary analysis with a descriptive and exploratory approach, dedicated exclusively to the evaluation of undergraduate medical programs in Brazil. It adopts a qualitative-quantitative design based on public data and academic reports, aiming to examine the 2023 ENADE (10) results for Medicine and the components used to calculate the PCC, identifying methodological limitations and inequalities among different types of institutions.

### Data sources

The study used multiple public sources of information. These included official INEP spreadsheets reporting 2023 ENADE results for Medicine (15) (Supplementary Data), including graduating students’ scores, IDOEP values, and continuous PCC scores. It also drew on the official ENADE student perception questionnaire, administered by INEP, which includes standardized items evaluating pedagogical organization, infrastructure, and training opportunities. Additionally, institutional performance reports and spreadsheets used in the PCC calculation were obtained from the INEP Open Data Portal and official public repositories of the Ministry of Education.

### Data analysis procedures

Data were extracted and organized for Medical Programs, classifying programs by administrative category such as federal, state, and municipal public institutions, as well as private for-profit and non-profit institutions. This disaggregated classification for public institutions was adopted to reflect the marked heterogeneity in funding, governance structures, and academic support across Brazil’s public higher education sectors, which could be obscured in a simple public vs. private analysis. Indicators including ENADE scores, IDOEP values, and PCC results were compared to identify disparities and performance patterns across institution types. The PCC formula for Medicine was examined in detail, considering its components and weights: ENADE score (20%), IDOEP (35%), faculty profile (30%), and student perception (15%). Finally, the study reviewed the instruments and procedures used for evaluation, highlighting methodological limitations identified in technical documents and academic reports.

### Interpretation strategy

Data were interpreted descriptively and comparatively to highlight inequalities among Medical Programs in Brazil. Subsequently, an analysis was conducted on the adequacy of the current model for assessing medical education quality.

### Ethical considerations

The research used only publicly available data and institutional reports without any identification of individual students or faculty members, ensuring adherence to applicable ethical principles.

### Statistics

Quantitative data were analyzed descriptively, with calculation of means, medians, standard deviations, and distribution of scores among higher education institutions (HEIs) offering medical programs. Comparisons were made by administrative category of HEIs (federal, state, municipal public institutions, and private for-profit or non-profit). Variations in PCC components were also examined, including ENADE scores (15), IDOEP values, faculty qualification and employment regime indicators, and student questionnaire results. Electronic spreadsheets were used for organizing and calculating descriptive statistics. Results were presented in tables and graphs to facilitate comparison among institution groups and to highlight regional and institutional disparities. Inferential or multivariate statistical techniques were not applied, as the data used are aggregated, census-type, and not derived from probabilistic samples. In such cases, methodological literature emphasizes that descriptive and exploratory analyses are the most appropriate approach, focusing on contextual interpretation rather than inferential generalization.

## Results

### General overview of ENADE 2023 in medicine

**Figure 1:**
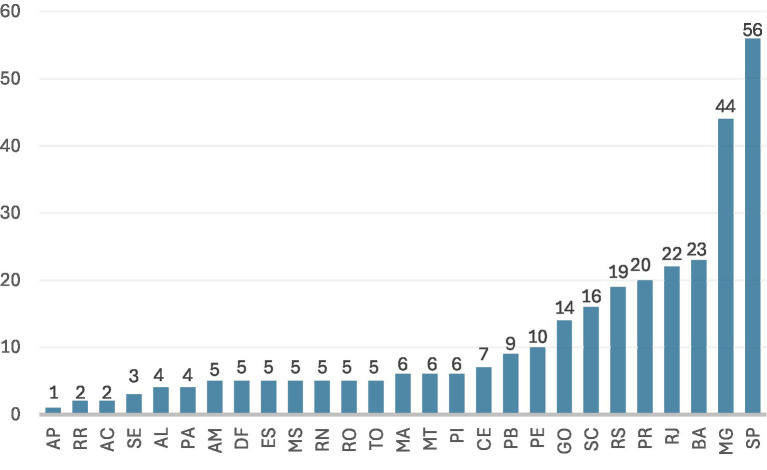
Number of medical schools by state (ENADE 2023). Number of medical schools by state (ENADE 2023). Distribution of the 309 medical schools evaluated by ENADE 2023 across Brazilian states. Values represent the number of schools in each state (14, 24). AP, Amapá; RR, Roraima; AC, Acre; SE, Sergipe; AL, Alagoas; PA, Pará; AM, Amazonas; DF, Federal District; ES, Espírito Santo; MS, Mato Grosso do Sul; RN, Rio Grande do Norte; RO, Rondônia; TO, Tocantins; MA, Maranhão; MT, Mato Grosso; PI, Piauí; CE, Ceará; PB, Paraíba; PE, Pernambuco; GO, Goiás; SC, Santa Catarina; RS, Rio Grande do Sul; PR, Paraná; RJ, Rio de Janeiro; BA, Bahia; MG, Minas Gerais; SP, São Paulo.

In 2023, Brazil’s estimated population was approximately 203 million. The 309 active medical schools evaluated by ENADE correspond to approximately 1.52 medical schools per million inhabitants. In the same year, about 31,054 students graduated, or approximately 153 graduating physicians per million inhabitants. We report these density indicators here for context; the figure itself depicts only the statewide distribution of schools (Figure 1). The PCC is calculated on a continuous scale from 0 to 5 and transformed into an ordinal concept (1 to 5) based on thresholds defined by INEP (15). Available data show substantial dispersion in results, with national means near the lower limit of the range for concept 4, but with unequal distribution across institution types.

The continuous PCC results (0–5 scale) for medical programs in 2023 revealed similar means across institutional categories but with differentiated distributions at the scale’s extremes. Federal and state public institutions presented a mean PCC of 2.78 ± 0.40, while private institutions showed an overall mean of 2.79 ± 0.45. Within the private sector, internal differences were evident: for-profit private institutions had a mean of 2.72 ± 0.48, while non-profit institutions reached 2.85 ± 0.42 (Table 1). The distribution by ordinal concept bands (1 to 5) showed that, among the top 100 PCC scores, approximately 62% were private institutions, particularly non-profit ones. In contrast, about 70% of courses with PCC 1 or 2 were for-profit private institutions. Public institutions were more frequently concentrated in the intermediate and higher bands.

**Table 1:** Number of medical programs by institutional category (ENADE 2023).

Category	Number of medical schools	CPC (Mean ± SD)
Federal public institutions	53	2.82 ± 0.38
State public institutions	37	2.74 ± 0.42
Municipal public institutions	23	2.65 ± 0.45
Private for-profit institutions	147	2.72 ± 0.48
Private non-profit	49	2.85 ± 0.42

Number of medical schools and average PCC scores (0–5) with standard deviation by institutional category (ENADE 2023, Brazil). PCC, Preliminary Course Concept.

### ENADE performance (graduate scores)

**Figure 2:**
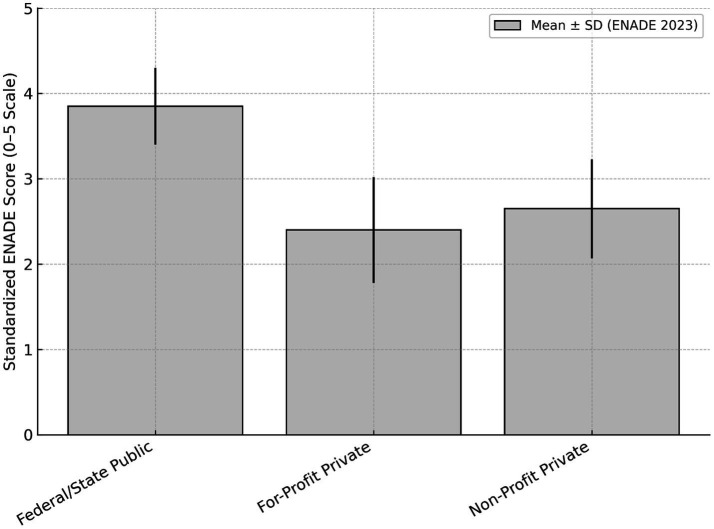
Standardized ENADE score (0–5) by institutional category. Mean ENADE scores (0–5 scale) with standard deviation shown by institutional category. Federal and state public institutions show higher average scores compared to private institutions.

The ENADE score (15), which accounts for 20% of the PCC calculation, showed consistent differences between public and private institutions in 2023. Federal and state public institutions had a standardized mean of 3.85 ± 0.45, while private institutions had a lower mean of 2.48 ± 0.60. Breaking down the private sector, for-profit institutions averaged 2.40 ± 0.62, and non-profit institutions reached 2.65 ± 0.58. This difference was also observed in internal test components. For general training, public institutions averaged 3.84 compared to 2.47 for private institutions. For specific knowledge (which accounts for 75% of the ENADE score), publics reached 3.70 versus 2.40 for privates. Some high-performing private universities were among the top national scores, with ENADE values ranging from 4.6 to 4.9. Figure 2 shows the standardized ENADE scores (0–5 scale) by institutional category. Federal and state public institutions presented higher mean ENADE scores compared to private institutions, while non-profit private programs performed slightly better than for-profit ones. The ENADE score represents 20% of the PCC calculation, while IDOEP accounts for 35% and student perception for 15%. Public institutions presented higher ENADE means, whereas private institutions presented higher averages in IDOEP and student perception.

### Indicator of Difference Between Observed and Expected Performance

**Figure 3:**
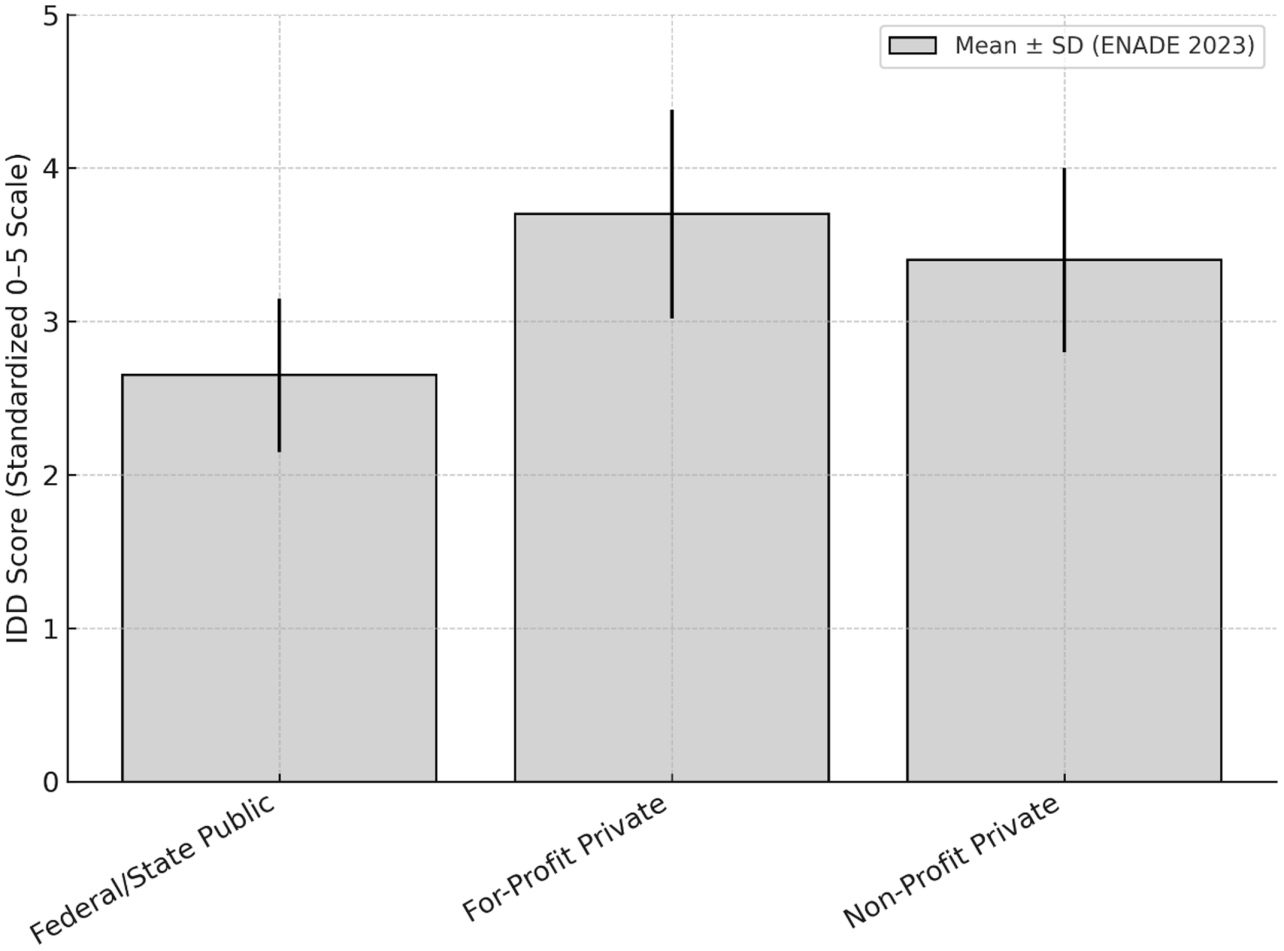
IDOEP score (0–5) by institutional category. Mean IDOEP scores with standard deviation for medical programs in Brazil (2023). The mean IDOEP score was 3.70 ± 0.68 for for-profit private institutions, 3.40 ± 0.60 for non-profit private institutions, and 2.65 ± 0.50 for federal and state public institutions. IDOEP, Indicator of Difference Between Observed and Expected Performance.

The IDOEP (15), which comprises 35% of the PCC, exhibited a pattern opposite to the raw ENADE score. Private institutions, especially for-profit ones, showed considerably higher IDOEP means: 3.70 ± 0.68 for for-profit private programs versus 3.40 ± 0.60 for non-profit ones. In contrast, federal and state public institutions had significantly lower averages of 2.65 ± 0.50. In distributional terms, over 60% of private programs achieved IDOEP scores ≥ 3.5, while only 20–25% of public programs reached this upper range (Figure 3).

### Faculty profile component

**Figure 4:**
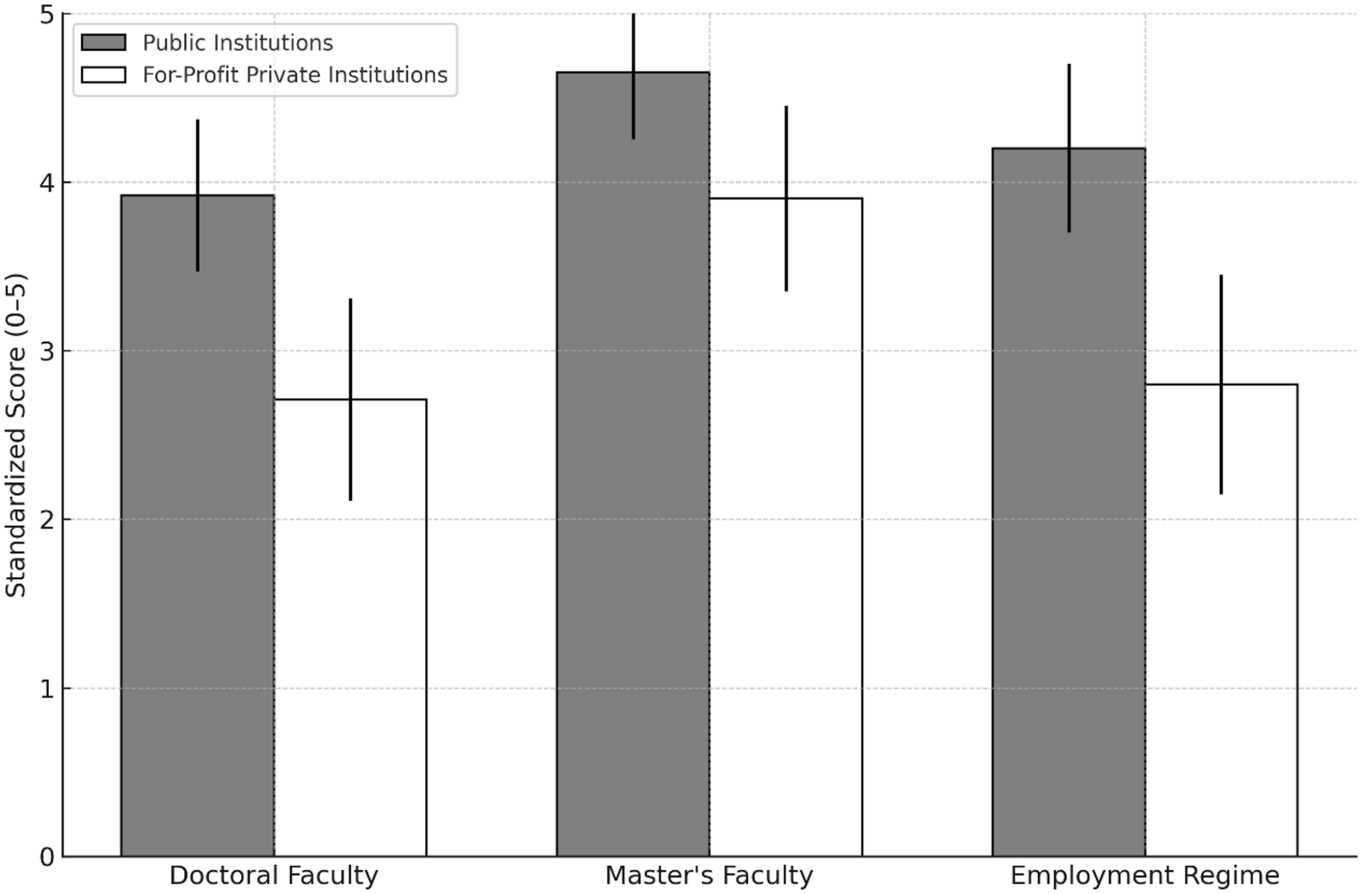
Faculty qualification and employment regime (0–5 Scale) by institution type. Mean standardized scores comparing public and for-profit private medical schools on doctoral and master’s degrees and faculty employment regime, showing higher values for public institutions.

The “Faculty Profile” component, which accounts for 30% of the PCC, revealed clear differences between federal and state public institutions and for-profit private institutions. On average, public institutions had a proportion of doctoral faculty of 3.92 ± 0.45, master’s-level faculty of 4.65 ± 0.40, and employment regime scores of 4.20 ± 0.50, indicating predominance of highly qualified faculty with full-time or exclusive dedication. Conversely, for-profit private institutions showed lower means in all indicators: doctoral faculty at 2.71 ± 0.60, master’s-level faculty at 3.90 ± 0.55, and employment regime at 2.80 ± 0.65. In the PCC calculation, the faculty profile component represents 30% of the score, while ENADE accounts for 20% and IDOEP for 35% (Figure 4).

### Student perception (student questionnaire)

**Figure 5:**
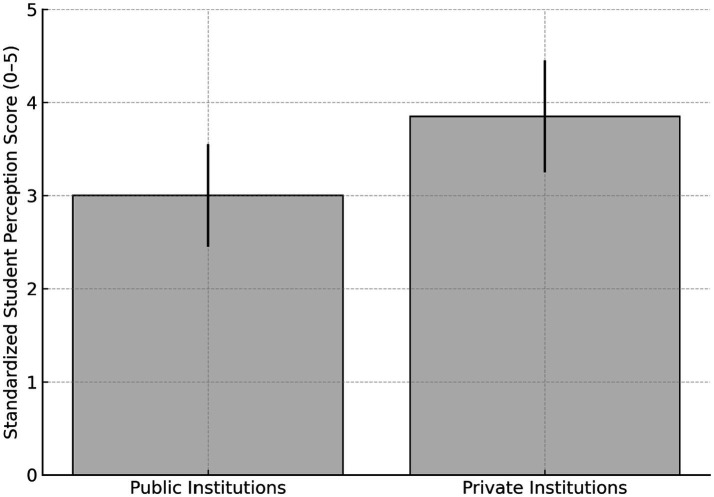
Student perception scores by institution type. Mean standardized scores (0–5 scale) with standard deviation for graduating student self-reported perception, comparing public and private medical schools in Brazil (ENADE 2023).

The “Student Perception” component, accounting for 15% of the PCC calculation, showed statistical differences between public and private institutions. The standardized mean for public institutions was 3.00 ± 0.55, while private institutions had a higher mean of 3.85 ± 0.60, indicating a consistent pattern of higher scores in this dimension (Figure 5) (15). Additionally, among the top 100 scores recorded on the student questionnaire, approximately 85% belonged to private institutions, with only 10 to 15 public programs appearing in this upper range. The standardized mean for student perception was 3.85 ± 0.60 in private institutions and 3.00 ± 0.55 in public institutions, based on a questionnaire administered to graduating students.

### Regional variation

**Figure 6:**
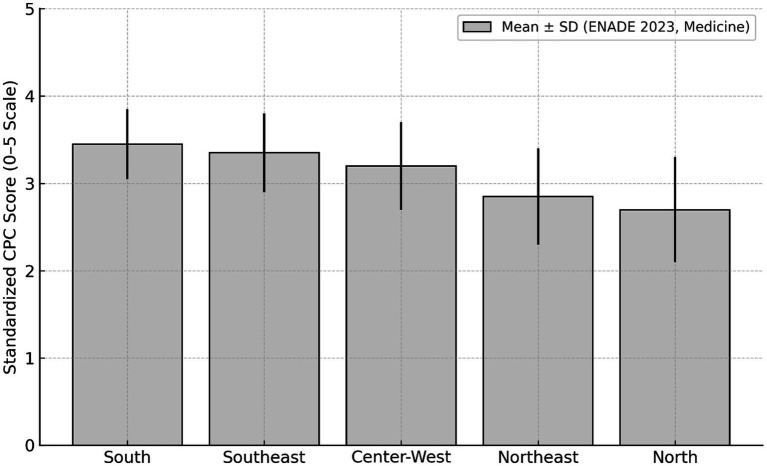
Regional disparities in mean PCC scores for medical schools (ENADE 2023). Mean standardized PCC scores (0–5 scale) with standard deviation for Brazilian regions (ENADE 2023, medicine). ENADE, National Student Performance Exam; PCC, Preliminary Course Concept.

Analysis of the 2023 ENADE data for Medicine (15) revealed significant regional disparities in PCC results. Standardized means of the continuous PCC (0–5 scale) varied consistently by region: the South had a mean of 3.45 ± 0.40, the Southeast 3.35 ± 0.45, while the Center-West averaged 3.20 ± 0.50, the Northeast 2.85 ± 0.55, and the North 2.70 ± 0.60. This variation was also reflected in the distribution of ordinal concepts (1 to 5): approximately 30–35% of programs in the South and Southeast achieved PCC 4 or 5 (considered good or excellent quality indicators), while around 45% of programs in the North and Northeast fell into the lowest bands (concepts 1 or 2). Even when limiting the analysis to federal public institutions, important differences persisted: federal programs in the South and Southeast had a mean PCC of 3.60 ± 0.35, while those in the North and Northeast averaged 3.05 ± 0.45. The standardized mean PCC scores were 3.45 ± 0.40 in the South, 3.35 ± 0.45 in the Southeast, 3.20 ± 0.50 in the Center-West, 2.85 ± 0.55 in the Northeast, and 2.70 ± 0.60 in the North (Figure 6).

### Synthesis of findings

Despite similar overall means between public and private institutions, there is greater internal inequality within the private sector, with both centers of excellence and many programs with insufficient scores. The current evaluation model (ENADE + IDOEP + student perception) favors programs admitting students with lower ENEM entrance scores (boosting IDOEP) and with strong perceived infrastructure, which can benefit certain private institutions. Regional and institutional disparities underscore the need for more context-sensitive evaluation, especially in designing public policies for regulation and support (Table 2).

**Table 2:** Mean PCC component scores by institution type.

Category	ENADE	IDOEP	Faculty profile	Student perception
Federal public institutions	3.85	2.65	4.26	3.00
State public institutions	3.85	2.65	4.26	3.00
Municipal public institutions	3.85	2.65	4.26	3.00
Private for-profit institutions	2.40	3.70	3.14	3.85
Private non-profit	2.65	3.40	3.82	3.85

Mean standardized scores (0–5 scale) for ENADE, IDOEP, Faculty Profile, and Student Perception components by institution type (ENADE 2023, Medicine). These values highlight differences in assessment dimensions across federal, state, municipal, and private medical schools in Brazil, reflecting institutional diversity and potential sources of inequality in PCC results. PCC, Preliminary Course Concept; ENADE, National Student Performance Exam; IDOEP, Indicator of Difference between Observed and Expected Performance; Faculty Profile, Average score combining doctoral proportion, master’s proportion, and employment regime; Student Perception, Mean standardized score from student self-reported questionnaire.

## Discussion

This paper seeks to contribute to the debate on the evaluation and regulation of medical education in Brazil by presenting a detailed analysis of the 2023 ENADE data for Medical Schools (15). By exploring national averages, disparities by administrative category and region, and the specific components of the PCC, the analysis provides evidence to rethink the current evaluation model (16).

ENADE was established as part of the National Higher Education Evaluation System (SINAES) with the aim of assessing graduating students’ performance in relation to curriculum guidelines, skills, and competencies (10, 11). In theory, it was intended to serve as a quality-inducing instrument, guiding regulation, oversight, and funding. However, studies show that over the years, ENADE has not systematically demonstrated its ability to promote concrete improvements or reduce inequalities in quality of Medicine Courses. This is partly due to the summative and punitive nature of the model, the limited formative use of results by institutions, and the weakness of mechanisms for monitoring and inducing change (17, 18).

Internationally, many countries require standardized licensing exams for all graduating medical students (e.g., USMLE in the United States (19), MCCQE in Canada (20), KMLE in South Korea (21)). However, these exams assess individual readiness for practice rather than serving as a regulatory ranking tool for institutions. Institutional evaluation relies on external reviews, on-site visits, and qualitative evidence. In contrast, the Brazilian ENADE (and its successor, ENAMED) aims to assess all graduating students but ties the evaluation focusing mainly on its own score and the IDOEP one. This structural difference raises concerns about fairness, validity, and the ability to accurately reflect educational quality across diverse contexts (22, 23).

Results reveal that although the overall means of the continuous PCC (0–5 scale) are very similar between public and private institutions (2.78 to 2.79), there is significant heterogeneity within these groups. Non-profit institutions had means comparable to or higher than federal public institutions (2.85 ± 0.42 vs. 2.82 ± 0.38), while state, municipal, and for-profit private institutions showed lower values. Moreover, the private sector included both centers of excellence (concept 5) and most of the Medical Programs classified as concepts 1 and 2, indicating a segmented and unequal landscape (24, 25).

Decomposition of PCC components highlighted important methodological limitations. The IDOEP, which weighs 35% in the final score, showed higher averages in for-profit private institutions (3.70 ± 0.68) than in public ones (2.65 ± 0.50). This difference stems from how IDOEP is calculated, as the difference between observed ENADE performance and that expected based on ENEM entrance exam score (15). Students in private programs generally have lower ENEM scores, which reduces the expected performance baseline and artificially inflates the measured added value, without adequately adjusting for socioeconomic factors (10, 26). Given that IDOEP has the highest individual weight in the PCC, this bias can significantly affect institutional rankings and incentivize market strategies focused on large-scale student recruitment.

To better reflect the curricular competencies defined by DCNs (12), the weight of the ENADE exam in the PCC calculation could be increased to 35–40%, ensuring that direct, standardized measures of student knowledge receive appropriate emphasis. Meanwhile, the IDOEP could be recalibrated to 25–30% using improved multilevel models that more fully adjust for socioeconomic and institutional factors. Such changes would align the Brazilian system with international best practices that prioritize direct assessments of competence for licensing (as in the USMLE or MCCQE) (19, 20) while still recognizing the value-added component. Statistical approaches like principal component analysis (PCA) could help validate the relative contributions of each metric, ensuring a more robust, fair, and context-sensitive assessment framework (22, 27).

Another important component, Student Perception (15% of the PCC), showed higher means in private institutions (3.85 ± 0.60) compared to public ones (3.00 ± 0.55), potentially reflecting investments in marketing and perceived infrastructure mostly in the earliest and pre-clinical semesters of the course. It is noteworthy that a considerable number of private institutions utilize high-standard public hospitals during the clinical cycle through contractual agreements (COAPES) (13). Therefore, in many private Medical Schools not only the lack of on-site visits in the evaluation process but also the absence of their own hospitals limits the PCC’s ability to capture important structural and pedagogical aspects (7, 8). In the United Kingdom, the General Medical Council (GMC) conducts regular inspections of Medical Schools, using a multi-method approach that includes document review, faculty interviews, and on-site observations (23). While the National Student Survey (NSS) provides valuable student feedback on course quality, it is treated as a qualitative input rather than a direct regulatory score (15). Student perceptions serve as an early warning system, triggering deeper audits or targeted reviews if issues are flagged (23). In Portugal, national reflections on the profile of recently graduated physicians also emphasize the need for strong integration between education and health systems, similar to the intentions of Brazil’s DCNs, highlighting the importance of community-based training and interdisciplinary competencies (28). By contrast, the Brazilian system currently assigns a fixed 15% weight in the PCC calculation to a single, self-reported student questionnaire administered only to graduating students, without complementary on-site visits or external validation. This design risks institutional strategies that optimize student satisfaction scores without necessarily ensuring robust educational quality or addressing structural deficiencies.

Regional analysis confirmed historical inequalities: the South (3.45 ± 0.40) and Southeast (3.35 ± 0.45) had higher means than the North (2.70 ± 0.60) and Northeast (2.85 ± 0.55), even among federal institutions. These differences reflect disparities in funding, teaching infrastructure, and human resources, consistent with evidence highlighting the difficulty of retaining professionals in less developed regions (24, 29).

Beyond these methodological issues, recent literature highlights the need for more effective government regulation to address the impacts of Brazil’s accelerated and uneven expansion of medical education, currently with 448 active Medical Schools (System e-MEC) (30, 31). National experience shows that although public policies such as the More Doctors Program have succeeded in increasing the number of courses and slots, especially in the private sector, this expansion has not always been accompanied by robust mechanisms for quality monitoring and equity. Studies show that unregulated growth, particularly of for-profit private schools, is associated with lower teaching quality, excessively large classes, less qualified faculty, and greater regional and social inequalities in the distribution of graduates (9, 24, 29). Furthermore, most professors with master’s or doctoral degrees are, at the same time, employed by public medical schools, where most postgraduate academic programs are located in Brazil. They are hired by private Medical Schools as hourly or even part-time teachers, without any significant institutional commitment. Therefore, strengthening state regulation, with clear definition of minimum quality standards, systematic oversight, on-site inspections, more comprehensive and transparent evaluation instruments is essential to align the growth of the training system with the needs of the Unified Health System and the goals of reducing regional and social health inequalities.

In the Brazilian context, other studies indicate that Medical Schools with better ENADE performance are associated with factors such as smaller class sizes, presence of stricto sensu postgraduate programs, more highly qualified faculty, and longer institutional histories (32). These variables, currently not directly included in the PCC, suggest that educational quality requires long-term and strong investment in infrastructure, research, and faculty development. In Brazil almost 95% of scientific research is developed in public universities.

Additionally, the current model lacks mechanisms to systematically capture faculty perspectives on teaching conditions and institutional infrastructure. The absence of instruments that reflect teachers’ views on laboratory quality, teaching hospitals, primary care networks, and pedagogical resources limits the analysis of the real conditions of training (18). Likewise, fundamental elements such as investment in stricto sensu postgraduate programs, strengthening university hospitals, and integrating research and extension activities into undergraduate education are not consistently evaluated, even though they are essential for articulating teaching, service, and community and for developing socially committed health professionals (32–34).

Despite concentrating a significant portion of Brazil’s scientific output and postgraduate (stricto sensu) programs in health, the country’s top public medical schools have historically played a limited and poorly coordinated role in shaping national undergraduate policies. This lack of institutional leadership in discussions on curricular guidelines and evaluation models contributes to a disconnect between academic excellence and existing regulatory frameworks. In many instances, strategic decisions regarding medical education are heavily influenced by private sector interests or are based on diagnostic analyses that fail to reflect the realities of teaching in leading institutions. This political-academic void on the part of the most prestigious universities undermines the potential for building a more qualified, equitable regulatory system aligned with the practices already adopted by reference institutions in the training of critical, humanistic, and socially committed professionals for the SUS (34, 36).

Taken together, these aspects point to the need for a thorough review of the evaluation model, capable of incorporating broader, more participatory dimensions sensitive to regional and institutional contexts. Finally, reflecting on ENAMED (11) represents a strategic opportunity to overcome current distortions and move toward a fairer formative evaluation system evaluating that does not reproduce existing inequalities and biases. While standardized national metrics like the Preliminary Course Concept (PCC) provide valuable data for higher education regulation, they must be interpreted within the broader context of structural inequalities across institutions. Without contextual calibration, such indicators risk reinforcing existing disparities rather than promoting meaningful improvement. In particular, subjective components such as student perception may disproportionately influence PCC results, potentially favoring some private institutions or specific regions. However, given the limitations of the available data, this remains a hypothesis that requires further empirical investigation. This study reinforces the need for evaluation models that combine robust quantitative data with qualitative, locally informed insights, especially in systems as heterogeneous as Brazil’s. We advocate for a more equitable and formative approach to medical course evaluation, one that supports continuous improvement and aligns more closely with national health priorities and the principles of the Unified Health System (SUS).

## Data Availability

The original contributions presented in the study are included in the article/Supplementary material, further inquiries can be directed to the corresponding author.
