# Supplementary material for: Evaluating medical education in Brazil: analysis of the National Student Performance Exam 2023
Source: Front Med (Lausanne). 2025 Oct 23;12:1679924. doi: 10.3389/fmed.2025.1679924 (PMC12592803; doi:10.3389/fmed.2025.1679924)
Supplement: Supplementary file 2 [file Data_Sheet_1.PDF]

federal goverrnment	Standardized Score - General Component (FG)	Standardized Score - Specific Component (CE)	ENADE Score (Continuous)
Standardized Score - General Component (FG)	1	0.449	0.603
Standardized Score - Specific Component (CE)	0.449	1	0.984
ENADE Score (Continuous)	0.603	0.984	1

state government	Standardized Score - General Component (FG)	Standardized Score - Specific Component (CE)	ENADE Score (Continuous)
Standardized Score - General Component (FG)	1	0.795	0.875
Standardized Score - Specific Component (CE)	0.795	1	0.989
ENADE Score (Continuous)	0.875	0.989	1

municipal government	Standardized Score - General Component (FG)	Standardized Score - Specific Component (CE)	ENADE Score (Continuous)
Standardized Score - General Component (FG)	1	0.867	0.931
Standardized Score - Specific Component (CE)	0.867	1	0.989
ENADE Score (Continuous)	0.931	0.989	1

special	Standardized Score - General Component (FG)	Standardized Score - Specific Component (CE)	ENADE Score (Continuous)
Standardized Score - General Component (FG)	1	0.805	0.862
Standardized Score - Specific Component (CE)	0.805	1	0.995
ENADE Score (Continuous)	0.862	0.995	1

community-confessional	Standardized Score - General Component (FG)	Standardized Score - Specific Component (CE)	ENADE Score (Continuous)
Standardized Score - General Component (FG)	1	0.61	0.765
Standardized Score - Specific Component (CE)	0.61	1	0.977
ENADE Score (Continuous)	0.765	0.977	1

Private for-profite	Standardized Score - General Component (FG)	Standardized Score - Specific Component (CE)	ENADE Score (Continuous)
Standardized Score - General Component (FG)	1	0.752	0.852
Standardized Score - Specific Component (CE)	0.752	1	0.986
ENADE Score (Continuous)	0.852	0.986	1

private non-profit	Standardized Score - General Component (FG)	Standardized Score - Specific Component (CE)	ENADE Score (Continuous)
Standardized Score - General Component (FG)	1	0.776	0.862
Standardized Score - Specific Component (CE)	0.776	1	0.989
ENADE Score (Continuous)	0.862	0.989	1
